# Tumor Enucleation vs. Partial Nephrectomy for T1 Renal Cell Carcinoma: A Systematic Review and Meta-Analysis

**DOI:** 10.3389/fonc.2019.00473

**Published:** 2019-06-04

**Authors:** Congcong Xu, Caixiu Lin, Zhen Xu, Sheng Feng, Yichun Zheng

**Affiliations:** ^1^Department of Urology, The Second Affiliated Hospital of Zhejiang University School of Medicine, Hangzhou, China; ^2^Department of Neurology, The Second Affiliated Hospital of Zhejiang University School of Medicine, Hangzhou, China; ^3^Department of Urology, The First Affiliated Hospital of Zhejiang Chinese Medical University, Hangzhou, China

**Keywords:** renal cell carcinoma, tumor enucleation, partial nephrectomy, nephron sparing surgery, meta-analysis

## Abstract

**Purpose:** Tumor enucleation (TE) and partial nephrectomy (PN) have both become main treatment strategies for T1 renal cell carcinoma (RCC), despite the discrepancy between their safety margin. We performed a meta-analysis on all the relevant trials in order to compare the clinical efficacy and safety of TE with those of PN for RCC treatment.

**Methods:** In this meta-analysis, randomized controlled trials or retrospective studies were included if they compared TE and PN therapy in patients with localized renal cancer. The main outcomes extracted were perioperative data and post-operative outcomes. Subgroups for analyses were undertaken according to tumor size and duration of follow up. Data were pooled using the generic variance method with a fixed or random effects model and expressed as mean differences or odds ratios with 95% CI.

**Results:** A total of 13 studies containing 1,792 patients undergoing TE and 3,068 undergoing PN were identified. Our study showed that the patients received TE had significantly shorter operative time (MD = −28.46, 95% CI = −42.09, −14.83, *P* < 0.0001), less hospital day (MD = −0.68, 95% CI = −1.04, −0.31, *P* = 0.0003), less estimate blood loss (MD = −59.90, 95% CI = −93.23, −26.58, *P* = 0.0004) and smaller change in estimated glomerular filtration rate (fixed effect: MD = 4.66, 95% CI = 1.67, 7.66, *P* = 0.002), fewer complications (fixed effect: OR = 0.65, 95% CI = 0.50, 0.85, *P* = 0.001) compared with those received PN. However, there were no significant differences in terms of warm ischemic time, positive margin rates, recurrence rates and survival rates between the two groups. All the subgroup analyses presented consistent results with the overall analyses.

**Conclusions:** Our findings suggested that TE is not only less-traumatizing and beneficial for recovery, but also better for renal function protection. Moreover, it did not show the evidence of an increase relapse rate or mortality rate when compared with PN.

## Background

Renal cell carcinoma (RCC) is the thirteenth most common malignancy worldwide, with the estimated number of newly diagnoses being 63,990 in the United States in 2017 (1). Apart from that, it is the most common kidney cancer in adults, and the treatment usually involves the surgical removal of whole or part of the kidney. Nowadays, advanced techniques and devices provide the urologists with a safer and more effective solution, which is also the goal for the surgical treatment of RCC.

For kidney cancer with the tumor diameter < 7 cm (T1a-b), nephron sparing surgery (NSS) is becoming the optimal choice because of its advantage in preserving renal function (2). Approaches to NSS include partial nephrectomy (PN) and tumor enucleation (TE). PN has become the gold standard for T1 renal cancer treatment since 2006 (3). Although standard surgical procedure of NSS should follow a route of excising at least 1 cm (safety margin) exterior to the tumor to ensure a true negative margin and to decrease the risk of local recurrence, some studies have proved that the safety margin could be < 1 cm (4). Several researchers regarded TE as an alternative to PN in terms of renal tumor treatment (5, 6). This surgical technique involves the blunt dissection of the renal tumor along the plane between the capsule and the healthy renal tissue, without injuring any normal renal parenchyma.

Currently, a large number of meta analyses regarding the comparison of radical nephrectomy (RN) and PN have been reported (7, 8), but few are about the comparison of TE and PN. Minervini et al. (9) performed a meta-analysis comparing several oncologic outcomes following TE and PN for renal cell carcinoma, and demonstrated that TE was at least non-inferior to PN. However, post-operative complications, survival rates or perioperative data had not been studied. Whether TE is a better treatment than PN for RCC remains unclear.

Hence, we identified and reviewed the evidence from clinical trials comparing TE with PN in patients with RCC.

## Methods

This meta-analysis was conducted according to the Cochrane Handbook for Systematic Reviews and Interventions. Results were reported in accordance with the Preferred Reporting Items for Systematic Reviews and Meta-Analyses guidelines.

### Study Selection Criteria

The inclusion criteria were: (1) adult patients with T1 RCC (T1 is further divided into T1a, the tumor is 4 cm cross or smaller; T1b, the tumor is larger than 4 cm but not 7 cm; no regional lymph node metastasis; no distant metastasis) according to American Joint Committee on Cancer (AJCC) TNM classification (10); (2) randomized controlled trials or retrospective studies; (3) treatment including TE and PN. The exclusion criteria were: (1) non-original articles; (2) not meeting the rule of control; (3) not a comparison between TE and PN.

### Search Strategy

A systematic literature search of PubMed^1^, Elsevier ScienceDirect^2^, Embase^3^, MEDLINE^4^, Web of Science^5^, Cochrane Library^6^, and Clinical Trials^7^ were performed to identify randomized controlled trials and retrospective studies up to August 2018. The search strategy was as follows: 1#: renal or kidney or nephroid or “renal cell” or “hyper nephroid” or “collecting duct”; 2#: cancer or tumor or carcinomas or adenocarcinomas; 3#: 1# and 2#; 4#: enucleation and “partial nephrectomy” or “nephron sparing surgery”; 5#: 3# and 4#. The relevant references from studies were read for additional randomized trails that fulfilled the eligibility criteria.

### Data Extraction

**Figure 1:**
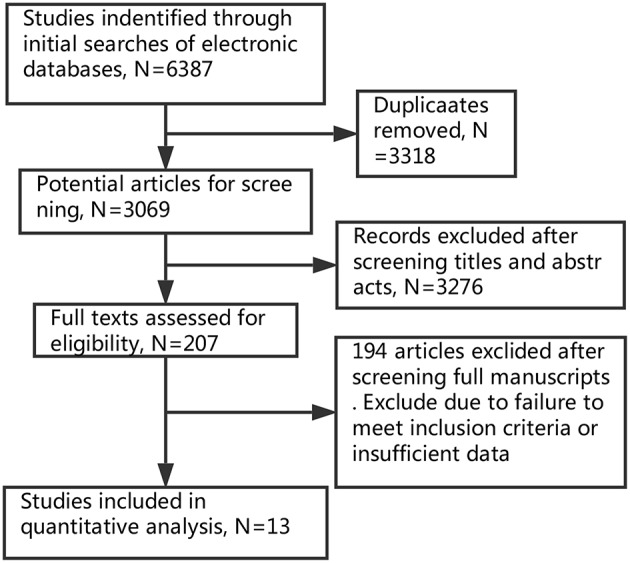
Flow diagram of literature search and study selection.

Two investigators independently screened the literature and extracted the information with the standard protocol. Disagreement was resolved until consensus was achieved. The flowchart of selection process is shown in Figure 1.

Extracted trial characteristics included: (1) title of article, first author's name and year of publication; (2) type of study design, method of randomization and duration of follow-up; (3) number of patients, tumor size and tumor stage. The outcome date included: (1) operative time, (2) length of hospital day, (3) warm ischemic (WI) time, (4) intraoperative estimate blood loss (EBL), (5) positive margins, (6) changes in estimated glomerular filtration rate (eGFR), (7) post-operative complications, (8) recurrence rate, (9) 5-year cancer specific survival (CSS), (10) 5-year progression-free survival (PFS).

### Quality Assessment

The quality of included retrospective cohort studies was assessed by means of the Newcastle-Ottawa scale (NOS) (Table 1) (11). The Cochrane's Collaboration (12) method was used to assess quality of randomized clinical trials (Table 2).

**Table 1:** Characteristics of 13 studies included in this meta-analysis.

**Studies**	**Year of publication**	**Type**	**Study quality**	**Mean FUP, months**	**TE group**			**PN group**		
					**Sample**	**Age**	**Tumor size (cm)**	**Sample**	**Age**	**Tumor size (cm)**
Longo et al. (13)	2014	Retrospective cohort study	7/9^*^	1	198	62.8	3.0	198	62.4	3.0
Minervini et al. (14)	2011	Retrospective cohort study	7/9^*^	120	537	61.8	3.3	982	60.1	3.4
Mukkamala et al. (15)	2014	Retrospective cohort study	6/9^*^	36	86	57	2.9	516	58	2.9
Cheng et al. (16)	2015	Retrospective cohort study	8/9^*^	60	20	54.2	3.22	12	70.2	4.78
Stephens et al. (17)	1990	Retrospective cohort study	5/9^*^	60	10	62	5.5	7	55	5.8
Wang et al. (18)	2017	Retrospective cohort study	6/9^*^	42	59	57.7	2.99	58	62.1	3.01
Lu et al. (19)	2017	Retrospective cohort study	7/9^*^	18	280	54.9	3.8	105	53	3.8
Calaway et al. (20)	2017	Retrospective cohort study	6/9^*^	/	13	/	3.2	34	/	2.9
Schiavina et al. (6)	2015	Randomized clinical trial	/	48	311	/	3.5	460	/	3.5
Snarskis et al. (21)	2017	Retrospective cohort study	8/9^*^	120	44	55	3.02	151	55	2.94
Huang et al. (5)	2016	Randomized clinical trial	/	18	44	51	2.65	45	52	3.0
Zhu et al. (22)	2017	Randomized clinical trial	/	23	119	56	2.8	127	54	3.1
Dong et al. (23)	2017	Retrospective cohort study	8/9^*^	12	71	58	3.0	373	61	3.3

FUP, follow up; TE, tumor enucleation; PN, partial nephrectomy;

* *Newcastle-Ottawa scale quality assessment*.

**Table 2:** Assessing the risk of bias of 3 RCTs.

**Study**	**Random sequence generation**	**Allocation concealment**	**Blinding of participants and personnel**	**Blinding of outcome assessment**	**Incomplete outcome data**	**Selective reporting**	**Other bias**
Schiavina et al. (6)	High risk	High risk	High risk	High risk	Low risk	Unclear risk	Unclear risk
Huang et al. (5)	Low risk	Low risk	High risk	High risk	Low risk	Unclear risk	Unclear risk
Zhu et al. (22)	Low risk	Low risk	High risk	High risk	Low risk	Unclear risk	Unclear risk

### Statistical Analysis

All calculations were performed using Review Manager 5.3 and STATA v12.0. The odds ratios (ORs) were computed to assess dichotomous data, and the mean difference was calculated for continuous data. Significant differences were affirmed if the exact 95% confidence intervals and the corresponding *P*-values were < 0.05. Outcome data were pooled for meta-analysis using a fixed-effect model when clinically appropriate and methodologically feasible to determine the summary effect of outcome data when insignificant heterogeneity was found. If significant heterogeneity was shown to exist, then a random-effects model was used for meta-analysis. The heterogeneity between the studies was evaluated by testing Cochran's Q-statistic and I^2^ statistic (*P* < 0.10 or I^2^ > 50 was defined as the heterogeneity significant). Subgroup analysis was undertaken according to follow-up time and tumor size, to assess the effect of varying outcome definitions.

Meanwhile, in order to test the reliability of the results, the sensitivity analysis was performed by sequential omission of individual studies. A study was considered influential when its removal changed the significance or significantly altered the overall heterogeneity. Furthermore, publication bias was analyzed by Begg's funnel plot and Egger's regression test (*P* < 0.05). If publication bias was suspected, then adjustment for funnel plot asymmetry was done by imputing missing study data using the Duval and Tweedie trim-and-fill method.

## Results

Based on our search criteria, 6,387 potentially relevant studies were identified and screened in primal search (Elsevier ScienceDirect: 1,153; Embase: 353; MEDLINE: 1,826; Cochrane Library: 12; Web of science: 3038; Clinical Trials: 5). All the papers were added into EndNote. After removing duplicates, 3,069 papers remained. The titles and abstracts were manually screened to determine if they met the inclusion criteria of this review. Finally, 13 studies (5, 6, 13–23) published from 1990 to 2017 met the inclusion criteria, and were analyzed. The thirteen studies contained 1,792 patients of TE and 3,068 patients of PN, but one study (24) was excluded eventually because no results were posted. Among 13 studies, there were three randomized controlled trials and ten retrospective studies. The main features of these studies are shown in Table 1.

### Perioperative Comparative Data

**Figure 2:**
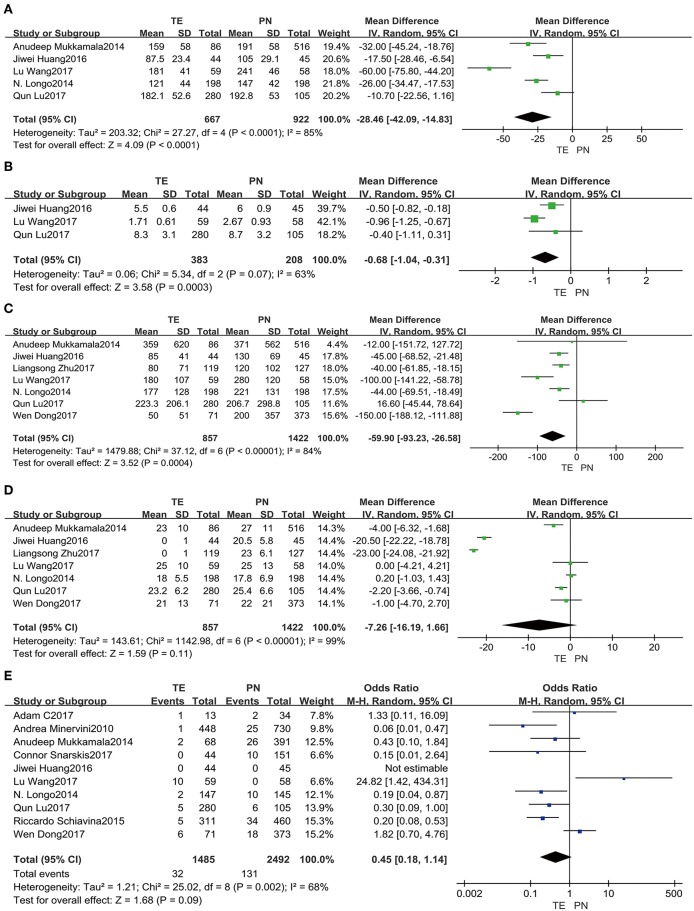
Forest plots of perioperative comparative data: operative time **(A)**, hospital day **(B)**, intraoperative EBL **(C)**, WI time **(D)**, positive margins **(E)**.

Operative time (Figure 2A) was assessed in the five studies (5, 13, 15, 18, 19). There were significant differences between TE and PN (fixed effect: MD = −25.77, 95% CI = −30.83, −20.71, *P* < 0.00001). Significant heterogeneity was indicated (*I*^2^ = 85%, *P* < 0.0001); and therefore, this comparison should be viewed with caution. As there was significant heterogeneity presenting, a random-effects model was used. It turned out that TE group spent a significantly shorter operative time than PN group (MD = −28.46, 95% CI = −42.09, −14.83, *P* < 0.0001).

Data pooled from the three included studies (5, 18, 19) reporting on hospital day (Figure 2B) showed a shorter time duration for TE group (fixed effect: MD = −0.73, 95% CI = −0.93, −0.52, *P* < 0.00001). Significant heterogeneity was also indicated (*I*^2^ = 63%, *P* = 0.07); so, this comparison should be viewed carefully. Using a random-effects model, it was confirmed that TE group had significantly shorter time duration in the hospital than PN group (MD = −0.68, 95% CI = −1.04, −0.31, *P* = 0.0003).

Intraoperative EBL (Figure 2C) from seven included studies (5, 13, 15, 18, 19, 22, 23) showed smaller amounts for TE group (fixed effect: MD = −55.66, 95% CI = −67.59, −43.73, *P* < 0.00001). This comparison should be viewed with prudence, because significant heterogeneity was revealed (*I*^2^ = 84%, *P* < 0.00001). A random-effects model was used as well, and it implied that TE group had a significantly less loss than PN group (MD = −59.90, 95% CI = −93.23, −26.58, *P* = 0.0004).

Statistics from the seven included studies (5, 13, 15, 18, 19, 22, 23) suggested that warm ischemic (WI) time (Figure 2D) was shorter for TE group (fixed effect: MD = −10.76, 95% CI = −11.37, −10.14, *P* < 0.00001). Using a random-effects model due to significant heterogeneity (*I*^2^ = 99%, *P* < 0.00001), there was no significant difference in WI time between the two groups (MD = −7.26, 95% CI = −16.19, −1.66, *P* = 0.11).

Positive margins (Figure 2E) were analyzed in the ten included studies (5, 6, 13–15, 18–21, 23), and it showed lower rates for TE group (fixed effect: OR = 0.44, 95% CI = 0.29, 0.65, *P* < 0.0001). Significant heterogeneity was showed (*I*^2^ = 68%, *P* = 0.002). Therefore, by a random-effects model, there was no significant difference between two groups (OR = 0.45, 95% CI = 0.18, 1.14, *P* = 0.09).

### Post-operative Outcomes

**Figure 3:**
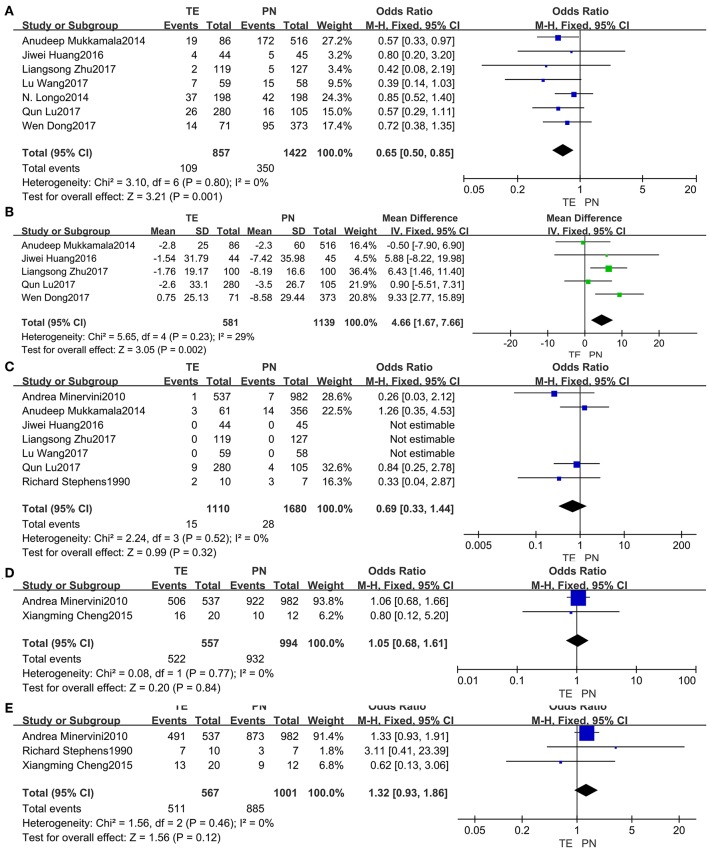
Forest plots of post-operative outcomes: post-operative complications **(A)**, change in eGFR **(B)**, recurrence rate **(C)**, 5-year CSS **(D)**, 5-year PFS **(E)**.

Data from the seven included studies (5, 13, 15, 18, 19, 22, 23) were analyzed, and it implied that post-operative complications (Figure 3A) were significantly less common in TE group (fixed effect: OR = 0.65, 95% CI = 0.50, 0.85, *P* = 0.001). And no significant heterogeneity was indicated (*I*^2^ = 0%, *P* = 0.80).

Changes in eGFR (Figure 3B) from the five studies (5, 15, 19, 22, 23) seemed more notable in PN group (fixed effect: MD = 4.66, 95% CI = 1.67, 7.66, *P* = 0.002). No significant heterogeneity was indicated (*I*^2^ = 29%, *P* = 0.23).

For the risk of recurrence (Figure 3C) from seven studies (5, 14, 15, 17–19, 22), no significant difference between the two groups was found (fixed effect: OR = 0.69, 95% CI = 0.33, 1.44, *P* = 0.32). No significant heterogeneity was indicated (*I*^2^ = 0%, *P* = 0.52).

Neither the 5-year CSS (Figure 3D) from the two studies (14, 16) nor the 5-year PFS (Figure 3E) from the three studies (14, 16, 17) was identified having significant differences between the two groups. There was no significant heterogeneity.

### Subgroup Analysis

**Figure 4:**
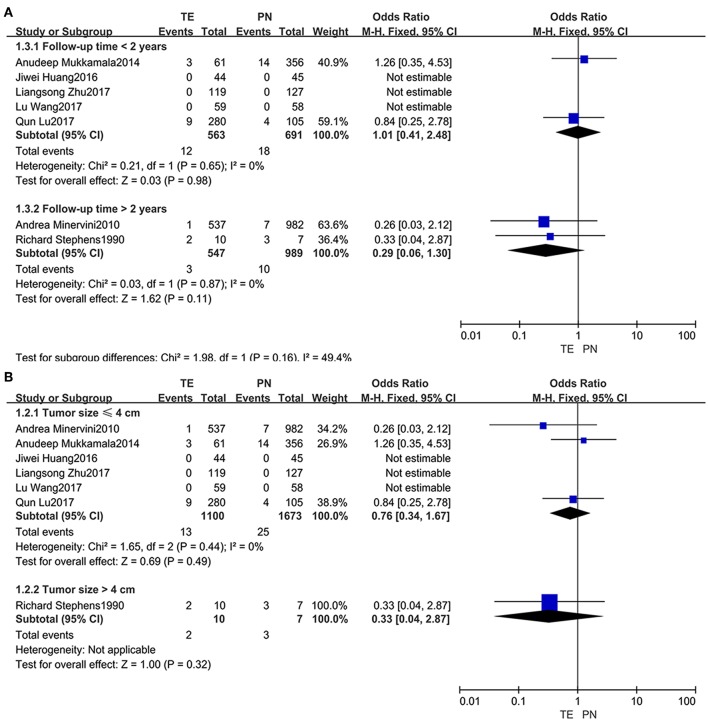
Forest plots of subgroups regarding recurrence rates: follow-up time **(A)**, tumor size **(B)**.

Subgroups were divided based on follow-up time (within 2 years or beyond) (Figure 4A) and tumor size (not larger than 4 cm or larger) (Figure 4B). When the subgroups regarding recurrence rates were compared, no significant difference was observed.

### Sensitivity Analysis and Publication Bias

**Figure 5:**
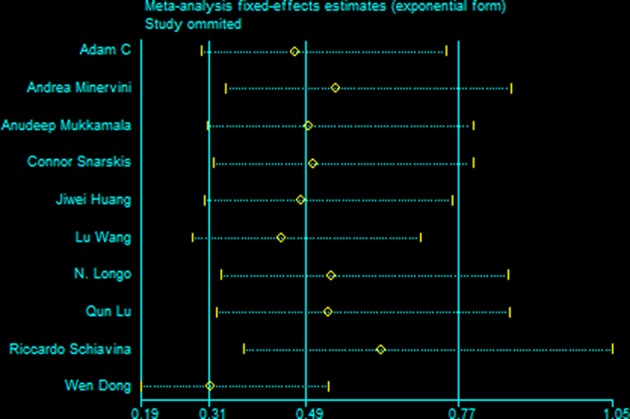
Results of an influence analysis in which the meta-analysis is re-estimated omitting each study in turn. Sensitivity analysis of instability in Positive Margins.

All studies were included in the sensitivity analysis, and one study was excluded at a time to explore the influence of each study on the overall outcome (Figure 5). No inconsistent results were found since we found the sensitivity analysis did not sufficiently change the stability and reliability of the results proved. Therefore, the sensitivity analysis indicated that our results were robust. Neither Begg's test and Egger's test results was significant (Table 3).

**Table 3:** Publication bias by Begg's test and Egger's test.

**Results**	**Operative time**	**Hospital day**	**EBL**	**WI**	**Positive margins**	**Complications**	**Recurrence**	**eGFR**	**CSS**	**PSF**
Begg	0.624	0.602	0.024	0.099	0.835	0.990	0.174	0.372	0.317	0.602
Egger	0.272	0.328	0.080	0.023	0.984	0.220	0.111	0.490	/	0.901

*EBL, estimate blood loss; WI, warm ischemic; eGFR, estimated glomerular filtration rate; CSS, cancer specific survival; PSF, progression-free survival*.

## Discussion

Nowadays, a growing number of clinical centers (5, 25, 26) preferred to use TE technique, but the trade-off between the advantages and the surgical morbidity remained debatable. Our meta-analysis involved 13 studies and reported several outcomes comparing clinical efficacy and oncology outcomes of TE and PN for RCC. The first concern with TE was positive margin rates. TE technique is defined as the blunt excision of the tumor without a visible margin, following the natural cleavage plane between the tumor capsule and the healthy parenchyma. Urologists still have one nagging doubt as to whether a visible margin increases the rate of positive margin or not. Kieran et al. thought that positive surgical margin would not increase the risk of local recurrence (27). On the other hand, in Kieran's study, all the patients with positive margins did undergo adjuvant flank radiotherapy, and thus the similar recurrence rates are likely due to the benefit of adjuvant radiotherapy instead of the surgical techniques. In our included studies, the rates of positive margins for TE and PN are 2.2 and 5.3%, respectively (5, 6, 13–15, 18–21, 23). However, our meta-analysis showed that there was no statistically significant difference. Therefore, TE does not increase the positive margin rates comparing PN.

In addition, many doctors also are concerned that TE may bring others safety related issues, such as tumor recurrence and a decreased post-operative survival. In our included clinical trials, the recurrence rates are 1.4 and 1.7% in TE and PN group, respectively (5, 14, 15, 17–19, 22); as for 5-year CSS, they are 93.7 and 93.8%, respectively (14, 16); for 5-year PFS, 90.1 and 88.4%, respectively (14, 16, 17). Similarly, the differences were not significant. All tumors in the included studies were in T1 stage according to TNM classification, and the tumor size ranged from 2.9 to 5.5 cm (minimum: 2.9 cm/maximum: 5.5 cm/median: 3.0 cm) (5, 14, 15, 17–19, 22). Furthermore, we found that the results of recurrence and survival rates did not change when the study (17) in which the tumor size was 5.5 cm was excluded. A large, retrospective, multicenter comparative study indicated that simple tumor enucleation had similar PFS and CSS rates (95.3 and 94.4%, respectively) compared to standard RN (28). Although tumor recurrence and patient survival yield a significant association with pathological grade or TNM stage (29), our findings indicated that neither of them was different between TE and PN method when the tumor was in T1 stage and its size was ~3.8 cm. In the subgroup analysis, no evidence could be confirmed that the tumor recurrence was directly relevant to follow-up time or tumor size; however, there was a lack of literatures for subgroup analyses.

With the development of surgical techniques, it is a trend to preserve renal function as much as possible during NSS. TE is considered to be an NSS procedure, aiming at maximizing preservation of healthy parenchyma and reducing the incidence of complications when the tumor is in the cortex especially for patients with solitary kidney. In this study, we tried to evaluate clinical efficacy of TE and PN. It is now considered that reducing operative time, WI time, intraoperative blood and sparing nephron could help avoid damaging the residual renal parenchyma. In our included studies, the average operative time is 155 and 168 min in TE and PN group, respectively (5, 13, 15, 18, 19); for the intraoperative EBL, 182 and 259 ml, respectively (5, 13, 15, 18, 19, 22, 23); for the WI time, 18 and 24 min, respectively (5, 13, 15, 18, 19, 22, 23); for the post-operative complications, 13 and 59%, respectively (5, 13, 15, 18, 19, 22, 23); for the Change in eGFR, 2.2 and 5.2 (mL/min/1.73 mm^2^), respectively (5, 15, 19, 22, 23). Notably, the differences were significant. Therefore, in our study, better protection of renal function and better clinical efficacy was reported in TE. Some scientists believe that preserving renal function could prolong survival. Antonelli et al. preformed a retrospective analysis of 3,457 patients who underwent radical or partial nephrectomy for RCC, and reported that preserving renal function using NSS for RCC helped to improve cancer-related survival (30). Nonetheless, Prof. Antonelli et al. acknowledged that the association of renal function with prognosis after cancer surgery was not intuitive, and that the underlying causes were difficult to deduce. In our study, better protection of renal function in TE was reported, but it had no significantly difference between TE and PN for cancer-related survival. The EAU Guidelines on Renal Cell Carcinoma also mentioned that TE was comparable in CSS and PFS rates to PN and RN (2). At present, the relationship between survival rate and renal function after RCC surgery remains controversial.

In the meantime, some reports regarding TE for high PADUA or RENAL scores laparoscopically and robotically have come to the fore in recent years (25, 31). Serni et al. (31) believes TE was a better option than PN for highly complex renal tumors, because complex renal tumors are often facing unfavorable nephrometry profiles. In our study, we could not compare the advantage between TE and PN for simple or complex renal tumors due to the lack of related controlled clinical trials.

At present, there are other similar meta-analyses. In the Minervini's review, the prevalence of positive surgical margins, loco-regional recurrence and renal recurrence in TE were higher than in PN (9). Whereas, most of their included studies did not compare between TE and PN at the same time, or just included the data which were exacted separately either for TE or PN groups but not both. There are inhomogeneity and bias with regard to study design. Therefore, their results remained controversial. Beyond that, Cao et al. (32) performed a meta-analysis and reported that TE had acceptable early oncology outcomes compared with traditional PN. However, their study had small samples and lacked in consideration on patient factors and tumor complexity.

Our inference is not devoid of limitations. First, the evidence level of included studies was generally low, because most included studies were retrospective analysis except for 3 RCTs. It is the largest hurdle in contemporary observational studies to adequately adjust for the inherent selection bias between the treatment groups. Besides, the conclusions (hospital day, 5 years-CSS and 5 years-PFS) were drawn on the basis of small study volume/number. Nevertheless, given the current existing data, the timing of this meta-analysis is appropriate.

## Author Contributions

CX, CL, ZX, SF, and YZ participated in data collection, manuscript drafting, table/figure creation, and manuscript revision.

### Conflict of Interest Statement

The authors declare that the research was conducted in the absence of any commercial or financial relationships that could be construed as a potential conflict of interest.

## Footnotes

^1^PubMed. Available online at: https://www.ncbi.nlm.nih.gov/pubmed

^2^Elsevier ScienceDirect. Available online at: https://www.sciencedirect.com/

^3^Embase. Available online at: https://www.embase.com/

^4^MEDLINE. Available online at: https://www.ncbi.nlm.nih.gov/pubmed

^5^Web of Science. Available online at: https://apps.webofknowledge.com

^6^Cochrane Library. Available online at: https://www.cochranelibrary.com/

^7^Clinical Trials. Available online at: https://www.clinicaltrials.gov/
